# Genome Wide Transcriptional Profile Analysis of *Vitis amurensis* and *Vitis vinifera* in Response to Cold Stress

**DOI:** 10.1371/journal.pone.0058740

**Published:** 2013-03-13

**Authors:** Haiping Xin, Wei Zhu, Lina Wang, Yue Xiang, Linchuan Fang, Jitao Li, Xiaoming Sun, Nian Wang, Jason P. Londo, Shaohua Li

**Affiliations:** 1 Key Laboratory of Plant Germplasm Enhancement and Specialty Agriculture, Wuhan Botanical Garden, The Chinese Academy of Sciences, Wuhan, P. R. China; 2 Beijing Key Laboratory of Grape Sciences and Enology, Laboratory of Plant Resources, Institute of Botany, The Chinese Academy of Sciences, Beijing, P. R. China; 3 Graduate School of Chinese Academy of Sciences, Beijing, P. R. China; 4 U.S. Department of Agriculture–Agricultural Research Service Grape Genetics Research Unit, Geneva, New York, United States of America; Wuhan University, China

## Abstract

Grape is one of the most important fruit crops worldwide. The suitable geographical locations and productivity of grapes are largely limited by temperature. *Vitis amurensis* is a wild grapevine species with remarkable cold-tolerance, exceeding that of *Vitis vinifera*, the dominant cultivated species of grapevine. However, the molecular mechanisms that contribute to the enhanced freezing tolerance of *V. amurensis* remain unknown. Here we used deep sequencing data from restriction endonuclease-generated cDNA fragments to evaluate the whole genome wide modification of transcriptome of *V. amurensis* under cold treatment. *Vitis vinifera* cv. Muscat of Hamburg was used as control to help investigate the distinctive features of *V. amruensis* in responding to cold stress. Approximately 9 million tags were sequenced from non-cold treatment (NCT) and cold treatment (CT) cDNA libraries in each species of grapevine sampled from shoot apices. Alignment of tags into *V. vinifera* cv. Pinot noir (PN40024) annotated genome identified over 15,000 transcripts in each library in *V. amruensis* and more than 16,000 in Muscat of Hamburg. Comparative analysis between NCT and CT libraries indicate that *V. amurensis* has fewer differential expressed genes (DEGs, 1314 transcripts) than Muscat of Hamburg (2307 transcripts) when exposed to cold stress. Common DEGs (408 transcripts) suggest that some genes provide fundamental roles during cold stress in grapes. The most robust DEGs (more than 20-fold change) also demonstrated significant differences between two kinds of grapevine, indicating that cold stress may trigger species specific pathways in *V. amurensis*. Functional categories of DEGs indicated that the proportion of up-regulated transcripts related to metabolism, transport, signal transduction and transcription were more abundant in *V. amurensis*. Several highly expressed transcripts that were found uniquely accumulated in *V. amurensis* are discussed in detail. This subset of unique candidate transcripts may contribute to the excellent cold-hardiness of *V. amurensis*.

## Introduction

Temperature is one of the primary environmental factors that influences and limits the growth and development of plant species. Low and freezing temperatures that occur in far northern and southern latitudes not only limits the suitable geographical locations for growing crops and horticultural plants, but also affects productivity [1]. Generally the cold-tolerant varieties of different species are used in conventional plant breeding as the main resource for increasing the freezing tolerance of cultivars. However, breeding for increased freezing tolerance without knowledge of the molecular processes responsible for cold hardiness traits limits the potential for crop improvement. Studies which examine signal transduction and gene expression changes during cold stress [1]–[6] not only help to reveal the sensing and regulatory mechanisms important for surviving low temperature stresses in plants, but also provide an approach to characterizing candidate genes for genetic improvement of freezing tolerance of agronomic plants [6].

Many plants show increased freezing tolerance following exposure to low nonfreezing temperatures, a phenomenon known as cold acclimation. During acclimation, plants sense environmental cues, e.g. cold signals, and reorganize their transcriptome in response. A series of biochemical and physiological changes then occur to protect plants from freeze-induced injury. While much is unknown about the actual perception of cold, recent studies indicate that plant cell membranes may play a major role in sensing decreasing temperatures. This perception results in transient increases in cytosolic Ca^2+^ levels, triggering a cascading biochemical and molecular reactions [3], [5], [7]. Abscisic acid (ABA) may also function as a secondary signal to transduce, at least in part, cold signal pathways [5]. Transcription factors also respond to cold signals at the early stage during low temperature exposure [8]. Perhaps the best example to date are the C-repeat binding transcription factors (CBF), which were identified as a key responders to low temperature stress in plants under cold stress [9]–[14]. The expression of CBF is up-regulated by another transcription factor, ICE1 (inducer of CBF expression 1)[15]–[19]. In the CBF-dependent pathway, the CBF protein recognizes the CRT/DRE cis-element in the promoter regions of COR (cold response) genes, which in turn activate transcription of these downstream genes and leads to chilling and freezing tolerance to plants. In a CBF-independent pathway, the transcription factors HOS10 (a R2R3 myeloblastosis type) play pivotal roles in the regulation of cold-responsive genes and freezing tolerance [20]. Although great progress has been made in recent years in understanding cold signal transduction in plant, the differences in genes or expression patterns in sensitive and tolerant species under cold stress still needs further investigation.

Grape is one of the most important and widely grown fruit crops in the world. The majority of cultivated varieties have been derived from one species, *Vitis vinifera*. Grapevine is cultivated for the production of fresh fruit, dried raisins, pressed for juice, or fermented to produce wine. Due to the popularity of this crop, grapevines are cultivated across broad geographic regions with many different climates. One of the primary limiting environmental conditions for grapevine cultivation is low temperature stress. If given sufficient time to acclimate to decreasing temperatures in the autumn, many *V. vinifera* cultivars can tolerate temperatures as low as −15°C in midwinter without suffering lethal injury [21], [22]. However, if temperatures drop too rapidly in late fall before acclimation occurs, or temperatures decrease below acclimated hardiness, serious injury can occur to buds and root tissues. Depending on the intensity and duration of freezing temperatures, partial or complete loss of fruit production can occur in the following year. In some cases, freezing temperatures can also result in damage severe enough to cause trunk damage and plant loss. Freezing tolerance of *V. vinifera* also drops quickly when buds break in early spring. As a result, early spring frost can damage floral primordia and reduce crop yields [22], [23]. Some wild *Vitis* species such as the North American species *V. riparia* Michx. and the Asian species *V. amurensis* can tolerate midwinter temperatures of −30°C and lower [22]. Thus these wild *Vitis* species have been used in grapevine breeding programs for the selection of new freezing tolerant cultivars. Most recently, studies of the molecular and genomic differences between these freezing tolerant species and freezing sensitive *V. vinifera* have been used to begin understanding the gene regulation of increased freezing tolerance. These studies may also provide candidate molecular elements for transgenic based genetic improvement.

Several *CBF* transcription factors have been identified in *Vitis*. Initially, three *CBF/DREB* family members were identified from *V. vinifera* and *V. riparia* [24]. Transcripts of *VvCBF 1-3* accumulated quickly after low temperature, drought and exogenous ABA treatments. *VvCBF4* was also identified from both *V. vinifera* and *V. riparia* [25]. The transcriptional level changes of VvCBF4 were seen to be maintained for several days, different from the transient expression of VvCBF1-3 [25]. No significant difference in expression pattern was observed between *V. riparia* and *V. vinifera*. [24], [25]. Expression level analysis shown that the *VvCBF4* was induced after 4 h at 4°C in leaf, stem and flower of *V. vinifera* cv. Koshu [26]. Transgenic lines of grape with over-expression of CBF and CBF-like genes; *VvCBF2*, *VvCBF4*, *VvCBFL* or *VvZFPL* (*V. vinifera* B-box-type zinc finger protein), show improved freezing tolerance without cold acclimation [21], [26]. In *V. vinifera* cv. Cabernet Sauvignon, the changes of transcript abundance in shoot tips when exposed to chilling (5°C) were evaluated by microarray analysis and the results indicate that chilling affects many calcium signaling transcripts [27].

Despite evidence that CBF genes and their transcriptional cascades are important for freezing tolerance, much about transcriptional regulation mechanisms related to cold acclimation are not clear. In this study, *V. amurensis* collected from Changbai Mountain (Jilin province in China) with high cold-hardiness was used to investigate responses to low temperature at a transcriptional level. A freezing-sensitive *V. vinifera* cv. Muscat of Hamburg was also examined as a control to evaluate the specific transcriptome modifications that occur in *V. amurensis* under cold stress. A well-established whole genome transcriptome analysis method, based on deep sequencing [28] and incorporating published grape genome sequences [29], was utilized to reveal the transcriptional changes that occur after cold treatment.

## Materials and Methods

### Ethics statement

No specific permissions were required for these locations/activities. This study did not involve endangered or protected species.

### Plants material and treatment

One-year-old self-rooted seedlings of *Vitis vinifera* cv. Muscat of Hamburg and *Vitis amurensis* (collected from Changbai Mountain in Jilin province in China) were grown and maintained in the greenhouse of Institute of Botany, Chinese Academy of Sciences, under a 16-h light/8-h dark photoperiod at 26°C in. Three days before cold treatment, seedlings were transferred into a chamber at 24°C under 16-h light at 6:00 am. Cold treatment was started at 9:00 am with constant light. During the first four hours, the temperature dropped 5°C per hour and was held at 4°C for an additional four hours. The seedlings that were used for control were also transferred into growth chambers but without cold treatment. The shoot apex with one well developed leaf was harvested from three independent replicates. RNAs were isolated for digital expression libraries construction and real-time RT-PCR analysis.

### Digital expression libraries construction and Solexa sequencing

Total RNA was isolated from collected samples with or without cold treatment, using plant total RNA isolation kit (Tiandz Inc; Beijing, China). The Gene Expression Sample Prep Kit (Illumina Inc; San Diego, CA, USA) was used for sequence tag preparation according to the manufacturer's protocol, which was also well described by Wu *et al*.[30]. Briefly, mRNA was purified with biotin-Oligo (dT) magnetic bead adsorption from 6 µg total RNA. First strand cDNA were synthesis with oligo (dT) on the bead. After second-strand cDNA synthesis, double strand cDNA was digested with NlaIII endonuclease, producing a bead-bound cDNA fragment containing sequence from the 3′-most CATG to the poly-A tail. These cDNA fragments were purified with magnetic bead precipitation and Illumina adapter 1(GEX adapter 1) was added to newly formed 5′ sticky end of cDNA fragments. The junction of GEX adapter 1 and CATG site was recognized by MmeI, which cuts 17 bp downstream of the CATG site, producing 17 bp cDNA sequence tags with GEX adapter 1. The 3′ fragments were removed with magnetic bead precipitation and Illumina adapter 2 (GEX adapter 2) was ligated to the new 3′ end of the cDNA fragment, which represented the tag library.

The cDNA fragments with GEX adapter 1 and 2 were undergoing 15 cycles of linear PCR amplification by Phusion polymerase (Finnzymes, Espoo, Finland). Resulted 85 base fragments were purified by 6% TBE PAGE Gel electrophoresis. After double strand denaturation, the single chain molecules were fixed onto the Solexa Sequencing Chip (flow cell). Each molecule grew into a cluster sequencing template through in situ amplification, which represented a single tag derived from a single transcript. Four color-labeled nucleotides were added during sequencing that performed by the Beijing Genomics Institute (BGI, www.genomics.org.cn) with Illumina HiSeq 2000 System. The data was submitted into the NCBI SRA database (Accession No. SRP018199). The produced 49 bp sequences contain target tags and 3′adaptor. Base-calling were performed using the Illumina Pipeline. After purity filtering and initial quality tests, the reads were sorted and counted for the following analysis.

### Sequence annotation

“Clean Tags” were obtained with Fastx-toolkit (http://hannonlab.cshl.edu/fastx_toolkit/) by trimming adapter sequences and filtering off adaptor-only tags and low-quality tags (containing ambiguous bases). Sequences alignments were carried out with Bowtie 0.12.8 [31] using Genoscope Grape Genome database (http://www.genoscope.cns.fr/externe/GenomeBrowser/Vitis/). VBI microbial database (http://vmd.vbi.vt.edu/) and BROAD institute database (http://www.broadinstitute.org/scientific-community/data) were used to exclude the contamination of tags from virus according to Wu *et al*.[30]. All clean tags were annotated based on transcript sequences of grape reference genes and masked grape genome sequences (exclude the repeating sequences). Only tags perfectly matched or 1 nt mismatch were considered for the further annotation. Sequences encoding proteins of known function were manually categorized into broad functional groups using the Munich Information Centre for Protein Sequences classification as guidance.

### Identification of differentially expressed genes

Annotated clean tags for each gene were calculated after alignment and then normalized to TPM (tags per million clean tags, [32], [33]). The genes that had TPM less than 10 in both library (NCT or CT) were excluded first. The default value (tag number) of genes that not found in one of the library was 1. Differentially expressed genes (DEGs) during cold treatment in two materials were identified based on a rigorous algorithm developed by Audic and Claverie [34]. P value was used to evaluate the authenticity of differential transcript accumulation [30], [34]. Bonferroni corrected p value was applied to control the FDR (false discovery rate) in the multiple comparison and analysis during the identification of DEGs [35]. An “FDR<0.001and the absolute value of log2-Ratio ≥1” was set as the threshold to determine the significance of gene expression difference.

### Real-time RT-PCR analysis

Samples were prepared and total RNA was isolated using the same method mentioned above. Real-time RT-PCR was carried out on three independent biological replicates each containing three technical replicates. First-strand cDNA was synthesized from RQ1 RNase-Free DNase (Promega, Madison, Wisconsin, USA)-treated total RNA using Superscript III reverse transcriptase (Invitrogen, Carlsbad, CA, USA) and diluted 20 fold as template. Specific primer pairs of selected genes were designed using Primer3 (v. 0.4.0, http://frodo.wi.mit.edu/) and shown in Table S2. Experiments were carried out using FastStart Universal SYBR Green Master (Roche Diagnostics, Mannheim, Germany) with SteopOneplus™ Real-Time PCR system (Applied Biosystems). Data were analyzed using qbasePLUS software (http://www.biogazelle.com/products). Transcript levels were normalized against the average of the grapevine reference genes: the UBC (ubiquitin-conjugating enzyme, EC922622) and GAPDH (glyceraldehyde 3-phosphate dehydrogenase, CB973647) according to Reid *et al*. [36]. The fold change in mRNA expression was estimated using threshold cycles, by the ΔΔCT method.

## Results

### Digital expression libraries construction and tag sequencing

The shoot apices with one well developed leaf from plant materials were collected and subjected to digital expression library construction. In order to activate plant cold stress responses and trigger changes in gene regulation, a total of 8 hours of cold treatment was used, with four hours gradient cooling from 24 °C to 4°C (the temperature dropped 5°C per hour) and another four hours held at 4°C. A total of four digital expression libraries were constructed from non-cold treatment (NCT) and cold treatment (CT) shoot apex of both *V. amurensis* and *V. vinifera* cv. Muscat of Hamburg.

For *V. amurensis*, 9,423,819 and 9,261,386 tags were sequenced from (non-cold treated) NCT and (cold treated) CT libraries, respectively (Table 1). After filtering out tags containing ‘N’ and adaptor sequences, tags were align to the VBI microbial database (http://vmd.vbi.vt.edu/) and BROAD institute database (http://www.broadinstitute.org/scientific-community/data) to exclude any contamination from viruses according to Wu *et al*. (2010). A total of 9,411,578 and 9,204,750 clean tags were collected from VaNCT and VaCT libraries. Single-copy tags in each library (291,588 in VaNCT and 264,143 in VaCT library) were excluded from further analysis [30]. Finally a total of 9,019,990 and 8,940,607 clean tags were clustered into 202,174 (VaNCT) and 205,283 (VaCT) unique tags. The distribution of unique tag with different copy number in each library were counted and shown in table 1.

**Table 1:** Tags in non-cold treatment (NCT) and cold treatment (CT) libraries in *V. amurensis* and *V. vinifera* cv. Muscat of Hamburg.

	*V. amurensis*		*V. vinifera* cv.	
			Muscat of Hamburg	
	NCT(24°C)	CT(4°C)	NCT(24°C)	NCT(4°C)
Total tag	9423819	9261386	9590554	9915588
Clean tag	9411578	9204750	9542864	9901892
clean tag copy = 1	291588	264143	57516	241889
unique Tag	202174	205283	182034	255879
unique tag copy number 2–5	126860	119128	39949	127105
unique tag copy number 6–10	25287	29270	47169	38836
unique tag copy number 10–20	16786	19810	36874	31561
unique tag copy number 21–50	14837	16996	30201	30064
unique tag copy number 51–100	7600	8403	14080	13954
unique tag copy number >100	10804	11676	13761	14359

For *V. vinifera* cv. Muscat of Hamburg, 9,590,554 and 9,915,588 tags were generated from NCT and CT libraries respectively (shown in Table 1). After filtering out low quality tags, contamination from viruses, and exclusion of single-copy tags, a total of 9,485,348 and 9,660,003 clean tags were clustered into 182,034 (NCT) and 255,879 (CT) unique tags. The higher number of unique tags observed in the VvCT library may indicate candidate genes related to cold signal response in Muscat of Hamburg. While the difference in unique tag number between cold treated and non-cold treated libraries (73,845 tags) was large in Muscat of Hamburg, the NCT and CT libraries in *V. amurensis* were much more similar and we identified only 3109 greater unique tags in the VaCT library (Table 1) However, this result is partially explained by the large number of unique tags with copy number between 2 and 5 (127,105 unique tags) in the VvCT library compared with the VvNCT library. These tags are the primary contribution to the significant increase in unique tags observed in Muscat of Hamburg during cold stress.

### The saturation of tags in each library

In order to increase the reliability of the expression analysis, the saturation of tags in each library was evaluated by the number of identified genes. The number of tags reached saturation when no new genes are detected. The results are shown in Figure S1. In the four libraries examined, the numbers of identified genes declined rapidly as the number of sequenced tags increased. All the libraries reached a plateau with 6 M tags. No new genes were identified as the tag number closed to 8 M. Considering that more than 9 M of the available tags were generated in each library, the tags were sequenced to saturation and provide a adequate information for the expression pattern analysis.

### Alignment of the unique tags to the reference genome

The unique tags from each library were aligned to the published genome and compared with annotated genes from *V. vinifera* cv. Pinot Noir PN40024 [29] (Table 2). Unique tags that matched the reference or had a single mismatch were counted. For *V. amurensis*, 150,435 unique tags (74.46%) in the VaNCT library and 161,448 unique tags (78.76%) in the VaCT library can be aligned to the reference genome. The proportion of aligned unique tags to reference genome in Muscat of Hamburg was higher than in *V. amurensis*, with 83.56% of the unique tags (151,981) in the VvNCT library and 84.86% of the unique tags (216,988) in the VvCT library. In *V. amurensis*, 88,872 unique tags (43.96%) in the VaNCT library and 99,522 unique tags (48.48%) in the VaCT library matched annotated reference genes. While in Muscat of Hamburg, 112,841 unique tags (61.99%) in the VvNCT library and 156,698 unique tags (61.24%) in the VvCT library were aligned to the ‘Pinot Noir’ annotated genes.

**Table 2:** Annotation of *V. amurensis* and *V. vinifera* cv. Muscat of Hamburg tags against the ‘Pinot Noir’ genomic sequences.

	*V. amurensis*		*V. vinifera* cv. Muscat of Hamburg	
	24°C (NCT)	4°C (CT)	24°C (NCT)	4°C (CT)
Unique tags match to genome	150435	161448	151981	216988
	74.46%	78.76%	83.56%	84.86%
Unique tags match to gene	88872	99522	112841	156698
	43.96%	48.48%	61.99%	61.24%
Matched genes	18118	18167	19211	20057
	74.42%	74.62%	78.91%	82.38%
Unitags match to one gene	79658	89522	100355	139921
	39.40%	43.61%	55.13%	54.68%
Matched genes	15549	15604	16188	17126
	63.87%	64.09%	66.49%	70.34%

For unique tags that only matched one gene, 39.40% of the unique tags in the VaNCT library and 43.61% of unique tags in the VaCT library were identified, while both libraries of Muscat of Hamburg shown higher percentage of aligned unique tags (55.13% in the VvNCT library and 54.68% in the VvCT library). These sets of data were used for the following gene expression pattern analysis. When compared with the total annotated genes in the 12× grape genome, both libraries in *V. amurensis* had similar identification results (15,549 genes in the VaNCT and 15,604 genes in the VaCT), while the VvCT library had slightly more matched genes (17,126, 70.34%) than the VvNCT library (16,188, 66.49%).

### Identification of differential expressed genes (DEGs) during cold treatment

**Figure 1:**
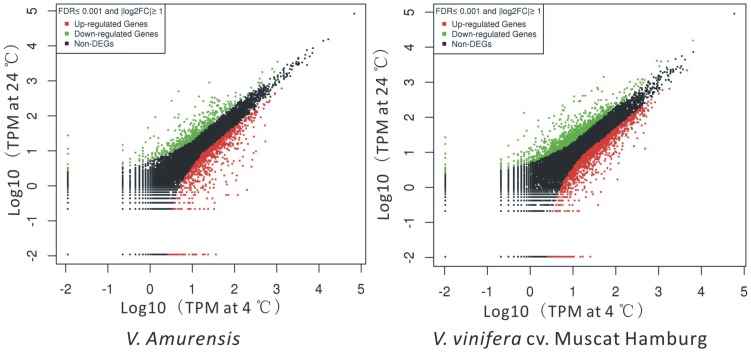
Identification of differentially expressed genes (DEGs) under cold treatment in *V. amurensis* and *V. vinifera* cv. Muscat of Hamburg. TPM (tags per million clean tags) were used to represent the expression levels of genes in non-cold treatment (NCT) and cold-treatment (CT) libraries in two grape varieties. Two parameters, “FDR <0.001” and “|log2 Ratio| ≥ 1” were used as the threshold to evaluate the significance of gene expression difference. Red and green dots represent the up- or down-regulated transcripts, respectively, during cold treatment in *V. amurensis* and Muscat of Hamburg. Black dots indicate transcripts without significant changes under cold stress.

Unique tags that perfectly matched reference genes in each library were normalized to tags per million clean tags (TPM) and used to evaluate the expression level of transcripts. To increase the robustness of the data, only the genes that had TPM more than 10 at least in one of the library (NCT or CT library) in each genotype were considered further. The transcripts with at least a two-fold difference in expression during cold treatment in the two genotypes (FDR <0.001) are displayed in Figure 1. Genes with less than two fold changes between NCT and CT libraries in both materials were excluded from further analysis. The details of DEGs, including original TPM, fold-change, annotation, P value and FDR in both materials are shown in Table S1.

The number of differentially expressed genes with at least a 2 or 5-fold change are shown in Table 3. The results indicate that *V. amurensis* has a lower number of DEGs during cold stress than Muscat of Hamburg. For *V. amurensis,* 1,314 genes changed at least 2-fold in the VaCT library, including 893 up-regulated genes and 421 down-regulated genes. In Muscat of Hamburg, 2,307 genes changed at least 2 fold in the VvCT library with 1,333 genes increasing and 974 genes decreasing in expression. A total of 167 and 77 genes were found to be up or down-regulated by at least five fold in the VaCT library, while the numbers increased to 174 and 149 in the the VvCT library. When comparing the DEG (at least 2 fold) between the two grape varieties, 429 genes showed the same expression pattern.

**Table 3:** Differential expressed genes (DEG) in *V. amurensis* and *V. vinifera* cv. Muscat of Hamburg during cold treatment.

		*V. amurensis*	*V. vinifera*	Common in both
DEG (at least **two** fold)	Total	1314	2307	428
	Up-regulated	893	1333	262
	Down-regulated	421	974	166
DEG (at least **five** fold)	Total	244	323	87
	Up-regulated	167	174	52
	Down-regulated	77	149	35

The DEGs with 20-fold or greater expression changes in both grapes are shown in Tables 4 and 5. Although less overall DEGs were found in *V. amurensis* than in Muscut of Hamburg, more genes (66) were up or down-regulated by more than 20-fold during cold treatment in *V. amurensis* (Table 4). Fifty-one genes were expressed at greater levels in the VaCT library. These genes could be grouped by gene ontology and were mainly associated with signal transduction (6), transcription (5), transport (4), protein folding (4), metabolism (7) and photosystem (6). Five transcription related genes belong to ERF, MYB and WRKY families, respectively. Cold regulated protein 27 (COR27, GSVIVT01009490001) was the highest up-regulated gene (332-fold) in the VaCT library. Fifteen DEGs were down-regulated greater than 20-fold in the VaCT library. Of these genes, 9 were related to protein folding (Shown in Table 5). GSVIVT01016426001 showed greatest decrease in expression (246-fold) in the VaCT library but has no annotation in the NCBI database.

**Table 4:** List of DEGs changed for 20 fold and more in *Vitis amurensis*.

Gene ID	Accession No.	Blastx results	*E*-value	Functional categorization	Fold
Up-regulated					
**GSVIVT01009490001**	NP_001031998	cold regulated protein 27 [Arabidopsis thaliana]	2.61E-40	stress related	332
**GSVIVT01025812001**	BAB85481	ACR toxin-sensitivity inducing protein [Citrus jambhiri]	5.04E-21	stress related	23
GSVIVT01011435001	XP_002514744	Calmodulin, putative [Ricinus communis]	1.13E-20	signal transduction	60
GSVIVT01027443001	XP_002266192	two-component response regulator [Vitis vinifera]	0	signal transduction	39
GSVIVT01025477001	XP_002283740	serine/threonine-protein kinase GSO1 [Vitis vinifera]	5.59E-161	signal transduction	29
GSVIVT01025105001	ABM67698	mitogen-activated protein kinase [Citrus sinensis]	0	signal transduction	25
GSVIVT01038710001	NP_851182	OBP3-responsive protein 1 [Arabidopsis thaliana]	0	signal transduction	21
GSVIVT01011652001	NP_001235886	circadian clock-associated FKF1 [Glycine max]	0	signal transduction	21
GSVIVT01013935001	XP_002282181	ethylene-responsive transcription factor 5 [Vitis vinifera]	4.66E-103	transcription	131
GSVIVT01013913001	XP_002281813	ethylene-responsive transcription factor 5 [Vitis vinifera]	2.54E-167	transcription	103
**GSVIVT01024916001**	NP_200765	myb family transcription factor [Arabidopsis thaliana]	6.78E-63	transcription	108
GSVIVT01015952001	ADU02585	WRKY transcription factor 4 [Vitis vinifera]	5.41E-180	transcription	68
GSVIVT01012682001	XP_002263115	WRKY transcription factor 6-like [Vitis vinifera]	0	transcription	30
GSVIVT01016284001	XP_002529577	Tyrosine-specific transport protein [Ricinus communis]	5.38E-121	transport	110
GSVIVT01023906001	XP_002528416	amino acid transporter, putative [Ricinus communis]	0	transport	51
**GSVIVT01007624001**	BAI63584	component of high affinity nitrate transporter [Lotus japonicus]	1.09E-86	transport	29
GSVIVT01027876001	XP_003590804	Peptide transporter PTR3-A [Medicago truncatula]	0	transport	21
**GSVIVT01026228001**	XP_003589717	Chaperone protein dnaJ [Medicago truncatula]	2.77E-167	protein folding	107
GSVIVT01020783001	XP_002525886	small heat-shock protein, putative [Ricinus communis]	6.17E-102	protein folding	76
GSVIVT01012660001	NP_567263	Chaperonin-like RbcX protein [Arabidopsis thaliana]	2.00E-70	protein folding	21
GSVIVT01014782001	XP_002530622	chaperone protein DNAj, putative [Ricinus communis]	4.21E-39	protein folding	20
GSVIVT01003728001	XP_002878742	serine carboxypeptidase S28 family protein [Arabidopsis lyrata]	0	proteolysis	25
GSVIVT01011355001	XP_002974500	serine carboxypeptidase-like enzyme [Selaginella moellendorffii]	1.63E-138	proteolysis	20
**GSVIVT01017521001**	XP_002530448	ubiquitin-protein ligase, putative [Ricinus communis]	0	ubiquitin	215
GSVIVT01009621001	XP_002871581	structural constituent of ribosome [Arabidopsis lyrata]	1.29E-37	translation	26
GSVIVT01022403001	ACR07827	ribulose-1,5-bisphosphate oxygenase large subunit [Linum volkensii]	7.82E-64	Metabolism	218
GSVIVT01010589001	ABJ97071	stilbene synthase 1 [Vitis vinifera]	0	Metabolism	97
GSVIVT01029173001	ACD03219	xyloglucan endotransglucosylase 9 [Actinidia hemsleyana]	3.71E-129	Metabolism	72
GSVIVT01028152001	XP_002515246	Glycerol-3-phosphate acyltransferase [Ricinus communis]	0	Metabolism	68
GSVIVT01013255001	XP_002527641	Flavonol synthase/flavanone 3-hydroxylase [Ricinus communis]	1.39E-148	Metabolism	53
GSVIVT01013272001	XP_002517513	Beta-amylase, putative [Ricinus communis]	0	Metabolism	32
GSVIVT01008169001	XP_002268089	UDP-glycosyltransferase 89B1-like [Vitis vinifera]	3.97E-39	Metabolism	29
GSVIVT01016698001	NP_565042	SAUR-like auxin-responsive protein family [Arabidopsis thaliana]	3.37E-28	hormome related	162
GSVIVT01037892001	NP_179101	indole-3-acetic acid-amido synthetase GH3.1 [Arabidopsis thaliana]	0	hormome related	31
GSVIVT01016441001	ADD30595	photosystem II protein D1 [Ehretia acuminata]	0	Photosystem	256
**GSVIVT01023733001**	XP_003622736	Photosystem I P700 chlorophyll a apoprotein [Medicago truncatula]	2.43E-24	Photosystem	180
**GSVIVT01017483001**	YP_567075	photosystem I P700 apoprotein A2 [Vitis vinifera]	0	Photosystem	149
GSVIVT01009295001	ACL81004	photosystem I subunit B [Gomesa lietzei]	1.53E-105	Photosystem	113
GSVIVT01029661001	YP_003330957	photosystem II protein D2 [Parthenium argentatum]	3.77E-47	Photosystem	98
GSVIVT01006662001	AAS60078	photosystem II CP43 protein [Medeola virginiana]	1.19E-102	Photosystem	23
**GSVIVT01009066001**	AAM64992	phi-1-like phosphate-induced protein [Arabidopsis thaliana]	1.55E-67	other	92
GSVIVT01009065001	AAM64992	phi-1-like phosphate-induced protein [Arabidopsis thaliana]	1.21E-89	other	33
GSVIVT01012636001	AFM35683	thiamin biosynthetic protein [Vitis pseudoreticulata]	0	other	47
GSVIVT01036062001	XP_002271636	CCR4-associated factor 1 homolog 9-like [Vitis vinifera]	2.96E-25	other	42
GSVIVT01015550001	NP_197519	tetratricopeptide repeat domain-containing [Arabidopsis thaliana]	6.28E-46	other	37
GSVIVT01013365001	XP_002270675	BTB/POZ domain-containing protein At2g30600 [Vitis vinifera]	0	other	21
GSVIVT01012794001				unknown	116
GSVIVT01013084001				unknown	86
GSVIVT01009359001				unknown	58
GSVIVT01019682001				unknown	48
GSVIVT01023736001				unknown	36
Down-regulated					
GSVIVT01038652001	NP_197841	downy mildew resistance 6 protein [Arabidopsis thaliana]	1.68E-179	stress related	24
GSVIVT01019655001	XP_002280048	homeobox-leucine zipper protein ATHB-12 [Vitis vinifera]	8.31E-139	transcription	21
GSVIVT01003927001	AAF20221	RNA-binding protein [Arabidopsis thaliana]	1.69E-30	RNA processing	47
GSVIVT01016572001	XP_003616269	15.7 kDa heat shock protein [Medicago truncatula]	8.18E-38	protein folding	22
**GSVIVT01016697001**	XP_003627410	17.4 kDa class III heat shock protein [Medicago truncatula]	1.50E-50	protein folding	108
GSVIVT01016567001	XP_002527736	heat shock protein, putative [Ricinus communis]	6.41E-176	protein folding	23
**GSVIVT01019407001**	BAD95030	heat-shock protein [Arabidopsis thaliana]	3.99E-142	protein folding	36
GSVIVT01028856001	XP_002513649	heat shock protein, putative [Ricinus communis]	0	protein folding	51
**GSVIVT01016429001**	ACZ48682	small heat shock protein 17.1 kDa [Vitis vinifera]	3.77E-70	protein folding	68
**GSVIVT01018654001**	CAA67022	LMW heat shock protein [Arabidopsis thaliana]	1.62E-46	protein folding	83
GSVIVT01021112001	XP_003593642	DnaJ homolog subfamily B member [Medicago truncatula]	5.70E-160	protein folding	23
**GSVIVT01007880001**	XP_002284179	chaperone protein ClpB1 [Vitis vinifera]	0	protein folding	103
**GSVIVT01017644001**	XP_002519290	Polygalacturonase precursor [Ricinus communis]	0	Metabolism	25
GSVIVT01000923001	XP_003621780	Bcl-2-associated athanogene-like protein [Medicago truncatula]	8.91E-09	other	66
**GSVIVT01016426001**				unknown	246

**Table 5:** List of DEGs changed for 20 fold and more in *Vitis vinifera* cv. Muscat of Hamburg.

Gene ID	Accession No.	Blastx results	*E*-value	Functional categorization	Fold
Up-regulated					
**GSVIVT01009490001**	NP_001031998	cold regulated protein 27 [Arabidopsis thaliana]	2.61E-40	stress related	241
**GSVIVT01025812001**	BAB85481	ACR toxin-sensitivity inducing protein [Citrus jambhiri]	5.04E-21	stress related	65
GSVIVT01034573001	NP_001238066	serine/threonine protein kinase-like protein [Glycine max]	1.04E-74	signal transduction	37
**GSVIVT01024916001**	NP_200765	myb family transcription factor [Arabidopsis thaliana]	6.78E-63	transcription	122
GSVIVT01018739001	ABI34654	bZIP transcription factor bZIP80 [Glycine max]	9.78E-94	transcription	33
GSVIVT01030258001	XP_002264974	WRKY transcription factor 33-like [Vitis vinifera]	0	transcription	26
GSVIVT01015538001	XP_003588760	Vacuolar transporter chaperone [Medicago truncatula]	9.40E-70	transport	38
**GSVIVT01007624001**	BAI63584	component of high affinity nitrate transporter [Lotus japonicus]	1.09E-86	transport	30
**GSVIVT01026228001**	XP_003589717	Chaperone protein dnaJ [Medicago truncatula]	2.77E-167	protein folding	141
**GSVIVT01017521001**	XP_002530448	ubiquitin-protein ligase, putative [Ricinus communis]	0	ubiquitin	28
GSVIVT01033920001	XP_002871114	40S ribosomal protein S17 [Arabidopsis lyrata subsp. lyrata]	6.58E-78	translation	48
GSVIVT01030086001	XP_002530221	glucose-6-phosphate 1-dehydrogenase, putative [Ricinus communis]	0	metabolism	95
GSVIVT01025303001	XP_003606776	Cytochrome c-type biogenesis protein ccmE [Medicago truncatula]	1.84E-35	metabolism	65
GSVIVT01012648001	NP_564785	glucose-6-phosphate/phosphate translocator 2 [Arabidopsis thaliana]	0	metabolism	24
GSVIVT01021978001	XP_002512299	shikimate dehydrogenase, putative [Ricinus communis]	0	metabolism	38
**GSVIVT01023733001**	XP_003622736	Photosystem I P700 chlorophyll a apoprotein [Medicago truncatula]	2.43E-24	photosystem	60
**GSVIVT01017483001**	YP_567075	photosystem I P700 apoprotein A2 [Vitis vinifera]	0	photosystem	21
GSVIVT01029087001	AEL98887	DNA primase large subunit, partial [Silene latifolia]	0	cell cycle	141
GSVIVT01010231001	XP_002272919	TIMELESS-interacting protein [Vitis vinifera]	0	cell cycle	99
GSVIVT01034539001	NP_564135	cupredoxin-like protein [Arabidopsis thaliana]	1.11E-55	other	160
**GSVIVT01009066001**	AAM64992	phi-1-like phosphate-induced protein [Arabidopsis thaliana]	1.55E-67	other	61
GSVIVT01016345001	XP_002523906	RNA-binding region-containing protein, putative [Ricinus communis]	1.42E-107	other	46
GSVIVT01010694001				unknown	23
**Down-regulated**					
GSVIVT01019830001	NP_001055135	Os05g0301600 [Oryza sativa Japonica Group]	3.19E-20	signal transduction	50
GSVIVT01035385001	AFI98399	heat shock transcription factor A2 [Vitis vinifera]	0	transcription	43
GSVIVT01019829001	ADC94861	HSP transcription factor [Vitis pseudoreticulata]	6.71E-164	transcription	261
**GSVIVT01007880001**	XP_002284179	chaperone protein ClpB1 [Vitis vinifera]	0	protein folding	21
**GSVIVT01016429001**	ACZ48682	small heat shock protein 17.1 kDa [Vitis vinifera]	3.77E-70	protein folding	21
GSVIVT01024994001	NP_001119156	heat shock 70kDa protein 1/8 [Arabidopsis thaliana]	1.85E-103	protein folding	23
GSVIVT01010308001	AAM96946	small heat shock protein [Solanum lycopersicum]	4.69E-83	protein folding	24
**GSVIVT01016697001**	XP_003627410	17.4 kDa class III heat shock protein [Medicago truncatula]	1.50E-50	protein folding	25
**GSVIVT01018654001**	CAA67022	LMW heat shock protein [Arabidopsis thaliana]	1.62E-46	protein folding	27
**GSVIVT01019407001**	BAD95030	heat-shock protein [Arabidopsis thaliana]	3.99E-142	protein folding	27
GSVIVT01017960001	XP_002516783	heat shock protein 70kD, putative [Ricinus communis]	0	protein folding	28
GSVIVT01026014001	XP_002526446	heat shock protein, putative [Ricinus communis]	0	protein folding	30
GSVIVT01003118001	XP_002517070	Heat shock factor protein HSF30, putative [Ricinus communis]	3.92E-109	protein folding	34
GSVIVT01016413001	ABF61863	chaperone [Agave tequilana]	1.88E-43	protein folding	37
GSVIVT01034195001	XP_002263599	heat shock cognate 70 kDa protein isoform 1 [Vitis vinifera]	2.31E-58	protein folding	38
GSVIVT01003939001	XP_003623314	Chaperone protein dnaJ [Medicago truncatula]	2.40E-176	protein folding	50
**GSVIVT01017644001**	XP_002519290	Polygalacturonase precursor, putative [Ricinus communis]	0	metabolism	21
GSVIVT01016417001	ACS87994	UDP-glucosyltransferase family 1 protein [Citrus sinensis]	1.29E-118	metabolism	22
GSVIVT01007681001	XP_003601214	Galactinol-sucrose galactosyltransferase [Medicago truncatula]	0	metabolism	40
GSVIVT01011397001	XP_002531859	Cell division protein ftsH [Ricinus communis]	0	cell cycle	52
GSVIVT01019816001				unknown	30
GSVIVT01001833001				unknown	35
GSVIVT01028075001				unknown	166
**GSVIVT01016426001**				unknown	184

Table 5 also shows other DEGs with large-fold changes (20-fold or more) in the VvCT library. Twenty-three genes were up-regulated in the VvCT library and these genes were associated with stress (2), transcription (3), transport (2), photosystem (2), cell cycle (2) and metabolism (4), etc. Three transcription factors belong to the MYB, bZIP and WRKY families, respectively. Twenty-four genes were expressed at greater than 20-fold lower levels in the VvCT library. Similar to the gene ontology observed in the VaCT library, most of the genes were related to protein folding (e.g. heat shock protein and chaperones).

Sixteen DEGs were identified that had 20-fold or greater changes in gene expression and were observed in both *V. amurensis* and *V. vinifera* (Table 5 and 6, marked by bold fonts). These genes include 9 up-regulated genes (GSVIVT01009490001, GSVIVT01026228001, GSVIVT01024916001, GSVIVT01025812001, GSVIVT01009066001, GSVIVT01023733001, GSVIVT01007624001, GSVIVT01017521001, GSVIVT01017483001) and 7 down-regulated genes (GSVIVT01017644001, GSVIVT01017644001, GSVIVT01016429001, GSVIVT01016697001, GSVIVT01018654001, GSVIVT01019407001, GSVIVT01016426001). It is interesting that the genes that had the greatest changes in expression in *V. amurensis* were the same genes identified in Muscat of Hamburg (shown in table 5). However, gene expression changes were less dramatic in the VvCT library. For example, COR27 was up-regulated 214 fold in Muscat of Hamburg and was up-regulated 332-fold in *V. amurensis*. Similarly, GSVIVT01016426001 was down-regulated 184-fold in Muscat of Hamburg and 246-fold in *V. amurensis*.

**Table 6:** Differential expressed transcription factors in *V. amurensis* and in *V. vinifera* cv. Muscat of Hamburg.

	Up regulated TF			Down-regulated TF		
TF	Va	Vv	common	Va	Vv	common
WRKY	15	15	9	1	1	0
AP2/ERF	10	12	5	1	4	1
MYB	9	13	4	6	10	3
Homeobox	7	3	0	4	5	2
NAC	6	4	3	0	3	0
C2H2L	6	6	5	0	1	0
bHLH	5	2	0	1	5	1
bZIP	3	3	1	2	6	2
trihelix	2	0	0	0	2	0
GRF	2	5	2	0	0	0
GRAS	2	2	0	0	4	0
MYC	1	0	0	0	0	0
Heat shock	1	0	0	3	2	1
ARF	1	3	1	0	0	0
Total	70	68	30	18	43	10

### Functional classification of cold stress-related DEGs

**Figure 2:**
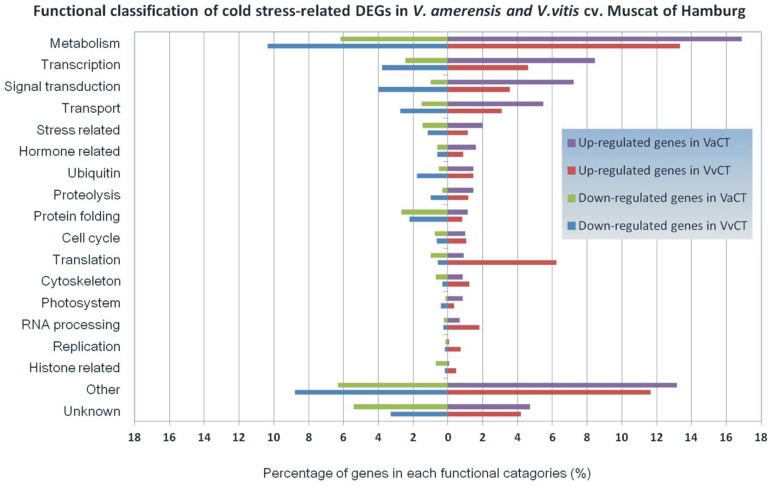
Functional classification of cold stress-related DEGs in *V. amurensis* and *V. vinifera* cv. Muscat of Hamburg. Identified DEGs were classified into functional categories and the percentage of up- or down-regulated functional categories were shown here. The DEGs with BLASTx annotation but that could not be classified into any of the functional categories were clustered into “Other”. Transcripts without any annotation information from BLASTx program were collected into “Unknown”.

The functional classification of DEGs was further examined in both grape varieties to investigate the pattern of transcriptome regulation that occurs during cold stress. The identified DEGs were annotated using BLASTN and BLASTX searches. Genes matching characterized proteins or proteins with putative functions were grouped according to functional categories (Figure 2). For up-regulated DEGs (Figure 2, right-hand side), genes encoding proteins involved in metabolism comprised the largest functional group in both grape varieties. In *V. amurensis*, transcription-related DEGs comprised the second largest category. A considerable proportion of the DEGs found in *V. amurensis* belong o signal transduction and transport categories. Compared with Muscat of Hamburg, a higher proportion of up-regulated DEGs related to metabolism, transcription, signal transduction and transport were found in *V. amurensis*. Translation and RNA processing related DEGs represented a lower proportion of DEGs in *V. amurensis*. The second largest category in Muscat of Hamburg was translation related genes (including ribosomal proteins). Genes related to transcription, signal transduction and transport also account for a large proportion of DEGs in Muscat of Hamburg.

Down-regulated DEGs in both CT libraries are shown in Figure 2 (Left-hand side). Many genes responsible for metabolism, transcription, signal transduction, transport and protein folding (including heat shock proteins and chaperones) were significantly down-regulated in both grape varieties. The proportion of genes with reduced expression that classified into translation, cytoskeleton, stress related, protein folding and histone related was higher in the VaCT than in the VvCT library.

Since the highest proportion of DEGs observed in *V. amurensis* were related to transcription, we further classified transcription factors (TFs) into gene families in both grape varieties (Tables 6). The TFs that showed the same trends in expression in both grape varieties are included in Table 6. A total of 70 up-regulated TFs were identified in *V. amurensis*, the majority of which included WRKY (15), AP2/ERF (10), MYB (9), Homeobox (7), NAC (6), C2H2L (6) and bHLH (5) families. Seven TF families including MYB (6), Homeobox (4), heat shock related (3) and others (5) were found down-regulated in *Vitis amurensis* (Table 6). Muscat of Hamburg had similar numbers of up-regulated TFs (68) and included the WRKY (15), AP2/ERF (12), MYB (13), C2H2L (6), GRF (5), and Homeobox (3) families. However, many more TFs were down-regulated (43) in Muscat of Hamburg, most of them belonging to the MYB (10), bZIP (6), Homeobox (5), AP2/ERF (4) and GRAS TF families. Three up-regulated TF families (trihelix, heat shock related and MYC) were found only in *Vitis amurensis* while four down-regulated TF families (NAC, C2H2L, trihelix and GRAS) were unique to Muscat of Hamburg.

### Validate the DEGs by real-time RT-PCR analysis

**Figure 3:**
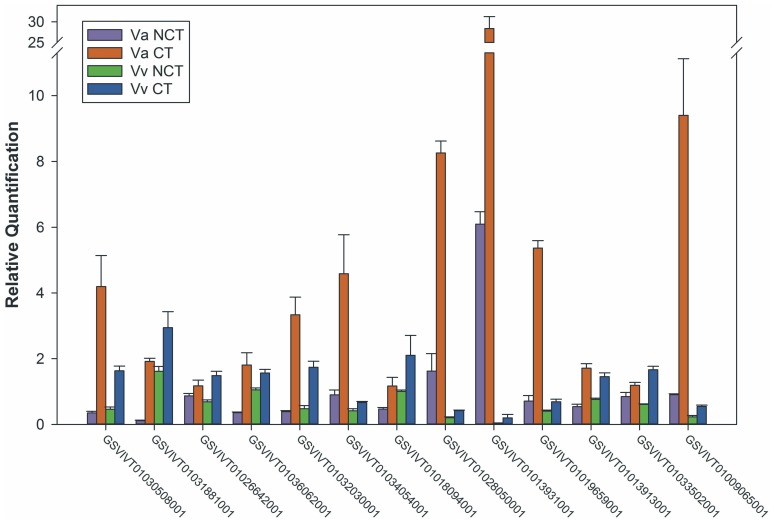
Real-time RT-PCR analysis for thirteen differentially expressed genes (DEGs) in *V. amurensis* and *V. vinifera* cv. Muscat of Hamburg. Real-time RT-PCR was carried out on three independent biological replicates each containing three technical replicates. The relative quantification of each transcript was normalized against UBC and GAPDH.

To validate the data from deep sequencing, thirteen up-regulated genes with a differential change in expression of 2 to 103 fold were randomly selected from *V. amurensis* DEGs for real-time RT-PCR analysis. The primers of selected genes are listed in Table S2. UBC and GAPDH were used as reference genes for data normalization according to Reid *et al*. [36]. The Real-time RT-PCR results in *V. amurensis* and Muscat of Hamburg are shown in Figure 3. The expression patterns of all detected genes show the same trend using RT-PCR and the Solexa-sequencing method. The list of the genes and the comparison of fold changes between deep sequencing and Real-time RT-PCR in two kinds of materials were shown in Table 7. The scale of the fold change of these genes based on Real-time RT-PCR was dramatically smaller (1–16 fold) than those of deep sequencing based method (2–103 fold, Table 7).

**Table 7:** List of genes selected for Real-time RT-PCR.

ID	Accession No.	Blasts results	*Vitis amurensis*		Muscat of Hamburg	
			Solexa fold	qPCR fold	Solexa fold	qPCR fold
GSVIVT01009065001	AAM64992	phi-1-like phosphate-induced protein [Arabidopsis thaliana]	**33**	**10**	**13**	**2**
GSVIVT01013913001	XP_002281813	ethylene-responsive transcription factor 5 [Vitis vinifera]	**103**	**3**	**3**	**2**
GSVIVT01013931001	XP_003634025	ethylene-responsive transcription factor 5-like [Vitis vinifera]	**13**	**5**	**9**	**5**
GSVIVT01018094001	XP_002284047	transcription factor UNE12 [Vitis vinifera]	**10**	**2**	**3**	**2**
GSVIVT01019659001	XP_002266775	transcription factor MYC2-like [Vitis vinifera]	**17**	**8**	**3**	**2**
GSVIVT01026642001	ADG58091	transcription factor [Lycoris longituba]	**2**	**1**	**3**	**2**
GSVIVT01028050001	ADP37419	ethylene-responsive-element-binding factor 4 [Petunia x hybrida]	**11**	**5**	**3**	**2**
GSVIVT01030508001	XP_002275341	1-aminocyclopropane-1-carboxylate oxidase 3 isoform 2 [Vitis vinifera]	**6**	**12**	**7**	**4**
GSVIVT01031881001	XP_002519124	GTP-binding protein alpha subunit, gna, putative [Ricinus communis]	**11**	**16**	**3**	**2**
GSVIVT01032030001	NP_198901	urophorphyrin methylase 1 [Arabidopsis thaliana]	**13**	**9**	**3**	**4**
GSVIVT01033502001	AEI60128	microsomal delta-12 oleate desaturase [Vitis labrusca]	**3**	**1**	**2**	**3**
GSVIVT01034054001	NP_001044811	Os01g0850000 [Oryza sativa Japonica Group]	**5**	**5**	**4**	**2**
GSVIVT01036062001	XP_002271636	CCR4-associated factor 1 homolog 9-like [Vitis vinifera]	**42**	**5**	**2**	**1**

## Discussion

### *V. amurensis* shown less sensitive to cold stress than Muscat of Hamburg at transcriptome level

In this study we used the Illumina HiSeq 2000 system, a high throughput DNA sequencing platform, to investigate the modification of the transcriptome under cold stress conditions in two grape varieties. Initially, we assumed that *V. amurensis*, one of the most cold-hardy wild grape species, may quickly response to cold stress and alter the regulation of its transcriptome intensively to overcome the potential for cold damage. In contrast we hypothesized that the cold sensitive variety, Muscat of Hamburg, may show less transcriptional level changes. However, deep sequencing results from cDNA libraries prepared from shoot apices with exposure to a total of 8 hours cold treatment did not support this hypothesis. While many differentially expressed genes were identified in the cold hardy *V. amurensis* in response to cold stress, a greater number of genes (2,307) were seen to increase in the cold sensitive Muscat of Hamburg.

One potential explanation for the lower number of DEGs identified in *V. amurensis* is a lack of alignment and annotation of *V. amurensis* genes to the reference genome. As one of the wild *Vitis* species, the phylogenetic distance between *V. amurensis* and PN40024 is larger than *V. vinifera* cv. Muscat of Hamburg and PN40024. Indeed, higher percentages of the unique tags were found to be aligned to reference genes in Muscat of Hamburg (55.13% in VvNCT and 54.68% in VvCT) than in *V. amurensis* (39.40% in VaNCT and 43.61% in VaCT library). The similar proportion of matched tags in *V. amerensis* were also found in previously published research [30], when transcriptome analysis was examined for *V. amerensis* before and after downy mildew infection ((35.47% in infected library and 36.99% in control). Because of the better alignment results, more genes were identified in Muscat of Hamburg than in *V. amurensis* (Table 2). In order to confirm that the reduced alignment ability of *V. amurensi*s is responsible for the differences in DEG number between the two kinds of grapes, we carefully checked the DEGs from Muscat of Hamburg in two *V. amurensis* libraries. The results show that almost all of the DEGs (2,271 from 2,307) in Muscat of Hamburg can be found in at least one of the library in *V. amurensis*. For *V. amurensis*, 1,309 DEGs out of 1,314 DEGs can be detected in at least one of the libraries in Muscat of Hamburg. These data indicate that the differences in DEG number between the two grape varieties are unlikely to be due to differences in the proportion of aligned tags. Instead, it suggests that there are very different modifications occurring in the transcriptome of these varieties during cold stress.

### *V. amurensis* responds to cold signal in a different way from Muscat of Hamburg

The differences observed in the transcriptome data, including DEG number, gene annotation and functional category, indicate that *V. amurensis* responds to cold signal in a different way from Muscat of Hamburg. For detected DEGs in *V. amurensis* and Muscat of Hamburg, very few genes (428 or 87 genes when two- or five-fold was used as threshold) show the same expression pattern during cold treatment (Table 3 and Table S1).

A considerable proportion of DEGs were up-regulated in *V. amurensis* compared with Muscat of Hamburg under cold stress treatments, suggesting that different genes are responding in the cold tolerant variety. For example, 68% of DEGs were found up-regulated in the VaCT library while 58% of DEGs were up-regulated in Muscat of Hamburg. Although less overall DEGs were identified in *V. amurensis*, the functional category analysis indicated that the VaCT library contained higher percentage of up-regulated DEGS involving in several biological processes including metabolism, transcription, signal transduction and transport (Figure 2). Meanwhile, a lower proportion of down-regulated DEGs in these four functional categories were observed in *V. amurensis* relative to Muscat of Hamburg. It is interesting that a large proportion of DEGs related to translation were identified in the VvCT library (Table 3), implying that Muscat of Hamburg may utilize the synthesis of new proteins as a mechanism for overcoming the damage of cold stress.

The genes which showed the most dramatic difference in expression in both CT libraries also show less similarity between the two genotypes (Table 4 and 5). For commonly changed genes, COR27 (GSVIVT01009490001) is a 27-kDa protein that responses to cold stress but no functional annotation in Arabidopsis is currently available [37]. Further studies indicated that COR27 is regulated by the circadian clock at warm growth temperatures and cold-induction of COR27 is gated by the clock [37]. Other genes like ACR toxin-sensitivity inducing protein (GSVIVT01025812001), myb TF (GSVIVT01024916001), nitrate transporter (GSVIVT01007624001) and chaperone protein dnaJ (GSVIVT01026228001), are also up-regulated in both kinds of grape. Most of these genes are involved in metabolism, transcription, transport, signal transduction and protein folding, and likely represent fundamental genes that respond to cold signals in *Vitis*.

Several of the genes which changed in expression in both grape varieties were seen to be expressed at much higher levels in *V. amurensis* than Muscat of Hamburg. GSVIVT01011652001 was annotated as circadian clock-associated FKF1, which may regulate the day-night rhythm during cold stress in *V. amurensis* and promote the expression of COR27. GSVIVT01013935001 and GSVIVT01013913001 are similar to ethylene-responsive TF, a type of protein related to CBF genes, that has been seen to be expressed in response to abiotic stress in plants [5], . GSVIVT01015952001 and GSVIVT01012682001 are annotated as WRKY TF. Genes in this family are related to several plant process including germination, senescence and responses to abiotic stresses such as drought and cold [40], [41]. Stilbene synthase 1(STS1, GSVIVT01010589001) was also up-regulated in the VaCT library. STS is widely studied in grape and it is the key enzyme that is responsible for the synthesis of resveratrol in biotic and abiotic stresses [42], [43]. It is interesting to observe that this important secondary chemical is expressed in response to cold stress in *V. amurensis*, potentially linking the biotic defense and antioxidant function of resveratrol, with damage to plant cells from low temperatures. GSVIVT01029173001 is homologous to xyloglucan endotransglucosylase, a gene related to cell wall modification and has also been observed to play a role in cold temperature response in Arabidopsis [44]. Beta-amylase (GSVIVT01013272001) was also seen to respond to temperature shock and lead to the accumulation of maltose [45].

### *V. amurensis* specific responses

In addition to these genes, a subset of highly expressed genes was only found only in *V. amurensis* (Table S1) and the majority of the genes relate to the cold stress response. These up-regulated genes are excellent candidate genes for cold stress response and may contribute to the higher cold-hardness of *V. amurensis* during winter.

#### Metabolism

The expression of 201 transcripts changed by more than 2-fold and were only observed in the VaCT library. Of these, nine transcripts show at least 5-fold up-regulation and contain more than 20 TPM in at least one of the libraries. Three transcripts were found to be related to starch synthase and degradation, including soluble starch synthase (GSVIVT01004632001), granule-bound starch synthase (GSVIVT01019680001) and alpha-amylase (GSVIVT01020069001). Starch syntheses are a group of important enzymes involved in the synthesis of amylose and amylopectin [46]. Normally, starch synthesis is under strong inhibition even under moderate water deficit condition [47] and none of members in this gene family were up-regulated in the VvCT library (Table S1). Amylase is responsible for hydrolyzing starch and glycogen into glucose and maltose. The increase of starch-degrading enzymes during stress condition, which leads to accumulation of soluble sugars, was also found in *Arabidopsis* [48]. Two transcripts were found to be related to sugar metabolism, including neutral invertase (GSVIVT01034944001) and glucose-6-phosphate 1-dehydrogenase (GSVIVT01000913001). In *Arabidopsis*, both mitochondrial and cytosolic invertase can generate glucose as a substrate for mitochondria-associated hexokinase, contributing to mitochondrial reactive oxygen species homeostasis [49]. Glucose-6-phosphate 1-dehydrogenase (G6PDH) is a key enzyme that regulates the flux of carbon through the pentosephosphate pathway. The transcript of G6PDH was accumulated during salt stress [50] and demonstrates a link between salt and cold stress response. Other robust up-regulated transcripts were annotated as UDP glycosyltransferase (GSVIVT01028812001), glutathione s-transferase (GSVIVT01024290001), carboxylesterase 1 (GSVIVT01010672001) and ATPase subunit 8 (GSVIVT01004977001). UDP glycosyltransferase mediates the transfer of glycosyl residues from activated nucleotide sugars to acceptor molecules [51]. And UDP-glucosyltransferase UGT74E2 in Arabidopsis mediates abiotic stress responses and stress-induced morphological adaptations by regulating auxin homeostasis [52]. Glutathione s-transferase not only functions in detoxification, but also responds to numerous stresses, including those arising from pathogen attack, oxidative stress, and heavy-metal toxicity in plant [53]. Carboxylesterase is an enzyme that is capable of hydrolyzing a wide variety of carboxylic acid esters. Although the functional details are still unknown, a member of this gene family in Arabidopsis (AT1G474800) was detected following cold treatment [54]. ATPase subunit is located in the membrane of mitochondria and constitutes the F0 complex of mitochondrial F-ATPases. The accumulation of ATPase subunit 8 in the VaCT library may indicate its potential roles in mitochondrial membrane stability and cold signal transduction.

#### Signal transduction

Nine genes that can be classified into signal transduction category were found more abundant (at least 5-fold up-regulated) in the VaCT library. Three of them were annotated as serine/threonine protein kinases (GSVIVT01024709001, GSVIVT01035226001 and GSVIVT01015198001). In plant cells, these kinds of kinases accept the information from receptors and convert it into appropriate outputs to regulate several biological processes [55]. A SNF1-type serine/threonine protein kinase of wheat was found to enhance multi-stress tolerance in Arabidopsis [56]. GSVIVT01024979001 and GSVIVT01021283001 were similar to receptor-like protein kinase (RPK), which localize in the cell wall and respond to the external challenges from environment [57]. RPKs that can response to cold stress was also identified in Arabidopsis [58]. GSVIVT01025028001 represents a ras-related protein, which belongs to small GTPase superfamily that participates in vesicular transport [59]. Mitogen-activated protein kinase-kinase-kinase (GSVIVT01022117001), part of the MAPK cascades involved in responses to various biotic and abiotic stresses, hormones, cell division and developmental processes in plant [60], was also up-regulated in the VaCT library. Together with OBP3-responsive protein 1 (GSVIVT01038710001) and choline/ethanolamine kinase (GSVIVT01001426001), these genes may take part in the cold signal transduction in *V. amurensis*.

#### Transcription

The DEGs in the transcription-related category were primarily transcription factors. For the 18 TFs that were up-regulated over 5-fold in *V. amurensis*, all of them had similar expression patterns in Muscat of Hamburg but with lower fold changes. These TFs may play fundamental roles for modifying the transcriptome during plant expose to cold environment. These TFs include ethylene-responsive transcription factor (GSVIVT01013935001, GSVIVT01013913001 and GSVIVT01013931001), WRKY transcription factor (GSVIVT01015952001, GSVIVT01021252001, GSVIVT01024624001 and GSVIVT01012682001), MYB family transcription factor (GSVIVT01024916001), AP2/ERF and B3 domain-containing transcription repressor TEM1-like (GSVIVT01011947001), DRE transcription factor 1 (GSVIVT01002262001), NAC transcription factor (GSVIVT01022354001), GRF domain class transcription factor (GSVIVT01038629001) and others. Members of TFs in these mentioned families were previously identified in response to cold signal and could regulate the expression of downstream genes in plant [8]. *V. amurensis* also triggers the up-regulated expression of distinct members in identified TF families including WRKY, AP2/ERF, MYB, Homeobox, NAC, *et al*. (Table 6). For example, six members of WRKY TFs (GSVIVT01011472001, GSVIVT01020136001, GSVIVT01030174001, GSVIVT01001332001, GSVIVT01028147001 and GSVIVT01034968001) were only up-regulated in the VaCT library (Table 6 and Table S1). WRKY TFs often act as repressors as well as activators for transcription, and members of the family play roles in abiotic stresses such as drought and cold [40]. Four AP2/ERF family genes (GSVIVT01016352001, GSVIVT01025100001, GSVIVT01020584001 and GSVIVT01035098001), were also identified with similar expression patterns. In Arabidopsis, CBF1 encodes an AP2 domain-containing transcriptional activator that binds to the C-repeat/DRE in response to low temperature and water deficit [10]–[12]. Other members of this gene family are also involved in abiotic stress responses [38]. Further investigation of the *V. amurensis*-specific TF in AP2/ERF and other TF family members will help to reveal the aspects of transcription that enable this species to tolerate cold stress.

#### Transport

Two transcripts related to transport were more abundant in the VaCT library, including a nitrate transporter (GSVIVT01008073001) and a hexose transporter (GSVIVT01003181001). The expression of a nitrate transporter gene NRT2 (At1g08090) was induced by cold, mannitol and NaCl treatment in the root of Arabidopsis [61]. Species specific expression differences of nitrate transporter were observed in citrus upon exposure to 4°C [62]. Very high transcript levels of nitrate transporter (431 fold increase) were observed in *Poncirus*, a cold hardy rootstock for citrus. While much lower levels were observed in *Satsuma mandarin* [62]. Higher transcription level of nitrate transporter gene indicates the possible role in nitrate acquisition in cold-hardness *V. amurensis*. A hexose transporter, VvHT5, was highly induced by powdery, downy mildew infection and wounding, suggesting its generalized response to stress in grape [63]. The key regulatory ABA biosynthetic gene, VvNCED1, was activated under these same stress conditions. The promoter region of VvHT5 contains multiple abscisic acid (ABA) response elements, suggesting a role for ABA in regulate the transcription of VvHT5 under stress conditions [63]. In our data, 6-fold up-regulation of hexose transporter (GSVIVT01003181001) was observed in the VaCT library with no obvious change in the VvCT library. And the expression of 9-cis-epoxycarotenoid dioxygenase 1 (NCED1, GSVIVT01038080001) was also up-regulated in both grapes (2.9-fold in VaCT and 2.2-fold in VvCT library, Table S1). Future investigation on the role of GSVIVT01003181001 and its regulation by ABA will help to understand the function of sugar metabolism related genes under cold stress.

#### Stress related genes

Two transcripts, GSVIVT01025994001 and GSVIVT01021103001, which were annotated as TMV (tobacco mosaic virus) resistance protein N-like and DEHYDRATION-INDUCED 19-like, respectively, were up-regulated only in *V. amurensis*. TMV resistance protein N encodes a TIR-NBS-LRR class protein that confers resistance to TMV [64]. The transcripts of *Dehydration-induced 19* in *Arabidopsis* are regulated by progressive drought stress in an ABA-independent manner [65]. Drought related protein may also respond to cold treatment as a result of the cross-talk between two stress signaling pathways in plants [66]. In fact, four DEGs, which related to drought stress (including AFG16868: dehydrin 1; BAD19956: drought-induced protein RDI; AAO33767: drought-induced protein and NP_177104: early-responsive to dehydration stress protein ERD4), were also found only in the VaCT library with 2 to 5-fold increases during cold treatment (Table S1). Other biotic and abiotic stresses related genes were also changed in VaCT, including disease resistance protein (GSVIVT01019041001, GSVIVT01005967001, GSVIVT01001043001 and GSVIVT01029297001), salt induced protein (GSVIVT01001340001, GSVIVT01010794001 and GSVIVT01029107001) and wound-responsive protein (GSVIVT01019074001 and GSVIVT01009760001). These genes may not only participate in the cold tolerance response but also play roles during the cross-talk between several stress-related networks in *V. amurensis*.

## Conclusion

The reorganization of the transcriptome during cold treatment in *V. amurensis* and *V. vinifera* cv. Muscat of Hamburg was evaluated by deep sequencing of short cDNA fragments. Overall, *V. amurensis* was seen to have less regulation of transcription profiles under cold stress than that of Muscat of Hamburg. However, the proportion of up-regulated DEGs involved in metabolism, transport, signal transduction and transcription were more abundant in *V. amurensis*. Additionally, several robust changes in transcript level were observed for candidate genes in *V. amurensis*. These genes may play an important role in the enhanced cold hardiness of *V. amurensis* and should be the focus of future studies in grapevine.

## Supporting Information

Figure S1 **Accumulation of uniquely mapped genes in non-cold treatment (NCT) and cold-treatment (CT) libraries in** ***V. amurensis*** **and** ***V. vinifera*** **cv. Muscat of Hamburg.**
(DOCX)

Table S1 **Detail information of DEGs in** ***V. amurensis*** **and** ***V. vinifera*** **cv. Muscat of Hamburg.**
(XLSX)

Table S2 **List of primers used for the Real-time RT-PCR.**
(DOCX)
