# Supplementary material for: Genome Wide Transcriptional Profile Analysis of Vitis amurensis and Vitis vinifera in Response to Cold Stress
Source: PLoS One. 2013 Mar 13;8(3):e58740. doi: 10.1371/journal.pone.0058740 (PMC3596283; doi:10.1371/journal.pone.0058740)
Supplement: Figure S1 — Accumulation of uniquely mapped genes in non-cold treatment (NCT) and cold-treatment (CT) libraries in V. amurensis and V. vinifera cv. Muscat of Hamburg. (DOCX) [file pone.0058740.s001.docx]

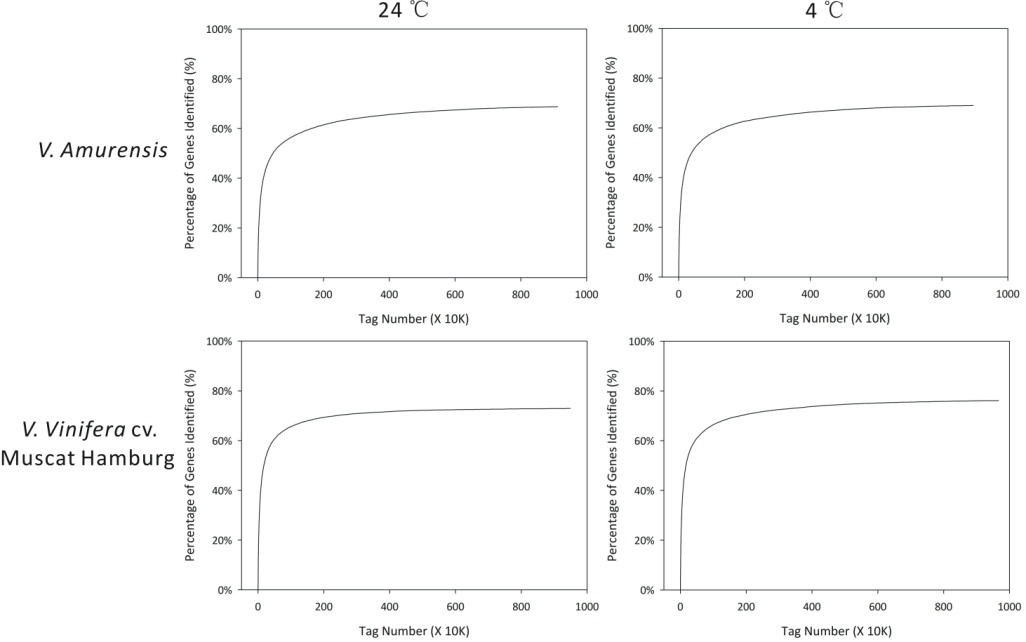


Figure S1. Accumulation of uniquely mapped genes in non-cold treatment (NCT) and cold-treatment (CT) libraries in *V. amurensis* and *V. vinifera* cv. Muscat of Hamburg. The gene numbers in all four libraries reached saturation when tag number closed to 8 M, and indicate there is adequate information for evaluate the gene expression pattern during cold treatment.
