# Supplementary material for: Genome Wide Transcriptional Profile Analysis of Vitis amurensis and Vitis vinifera in Response to Cold Stress
Source: PLoS One. 2013 Mar 13;8(3):e58740. doi: 10.1371/journal.pone.0058740 (PMC3596283; doi:10.1371/journal.pone.0058740)
Supplement: Table S2 — List of primers used for the Real-time RT-PCR. (DOCX) [file pone.0058740.s003.docx]

Table S2. List of primers used for the Real-time RT-PCR.

|  | **Forward primer** | **Reverse primer** | **Target size** |
| --- | --- | --- | --- |
| GSVIVT01009065001 | GTATGCTGGGGATTTGTTGG | TCATACAAGGCAGGAAGCAA | 96 |
| GSVIVT01013913001 | GATCTGATCCACCAGCGATA | TTCGGGCAAGTACTCCTCTT | 110 |
| GSVIVT01013931001 | CATTCTCAATTTCCCCCTTG | CTTCGGTCCCAAAATCTCTC | 95 |
| GSVIVT01018094001 | CTGCTATGCAGTTCCTCCAA | CTCAGGCTTGATGAGCGTAG | 110 |
| GSVIVT01019659001 | GGTTCAGTGTCCCGATCTAAA | TGAAGCATCAGCTCCTTGAC | 114 |
| GSVIVT01026642001 | CATGACTCCCATTTCTGATAACTC | GGCCGCTCCTGAAACTAAG | 120 |
| GSVIVT01028050001 | CTATCTCGACGGCAACTCAG | GAGGAGCAACTGCAAATTCA | 116 |
| GSVIVT01030508001 | AAACGTTGAAGCCAGTGTCA | ACACAACCTGGAAACAAGCA | 97 |
| GSVIVT01031881001 | TACCATCAAGGTGCAAAACG | AATTGGGCAGAGAAGGAGAA | 103 |
| GSVIVT01032030001 | TCACCGACACTGATCGTCAT | TCTATTTCGCCTCTACCAGGA | 97 |
| GSVIVT01033502001 | GGCTACAAAGGCCATAAAGC | CACCATCCGACTGGACATAG | 116 |
| GSVIVT01034054001 | TCCGGTTACCTCAAGAATGG | TCAATCCCTTCACCACTTCC | 118 |
| GSVIVT01036062001 | CGCGTACGATTTTGGGTACT | GTTCCGAAAAACACCCTCAG | 99 |
